# Novel mutations in the *COL2A1* gene in Japanese patients with Stickler syndrome

**DOI:** 10.1038/hgv.2016.18

**Published:** 2016-07-07

**Authors:** Hiroyuki Kondo, Itsuka Matsushita, Tatsuo Nagata, Takaaki Hayashi, Masashi Kakinoki, Eiichi Uchio, Mineo Kondo, Masahito Ohji, Shunji Kusaka

**Affiliations:** 1 Department of Ophthalmology, University of Occupational and Environmental Health, Kitakyushu, Japan; 2 Department of Ophthalmology, The Jikei University School of Medicine, Tokyo, Japan; 3 Department of Ophthalmology, Shiga University of Medical Science, Hikone, Japan; 4 Department of Ophthalmology, Fukuoka University, Fukuoka, Japan; 5 Department of Ophthalmology, Mie University Graduate School of Medicine, Tsu, Japan; 6 Department of Ophthalmology, Kinki University, Sakai Hospital, Sakai, Japan

## Abstract

Stickler syndrome is an inherited connective tissue disorder that affects the eyes, cartilage and articular tissues. The phenotypes of Stickler syndrome include congenital high myopia, retinal detachment, premature joint degeneration, hearing impairment and craniofacial anomalies, such as cleft palate and midline facial hypoplasia. The disease is genetically heterogeneous, and the majority of the cases are caused by mutations in the *COL2A1* gene. We examined 40 Japanese patients with Stickler syndrome from 23 families to determine whether they had mutations in the *COL2A1* gene. This analysis was conducted by examining each patient’s genomic DNA by Sanger sequencing. Five nonsense, 4 splicing and 8 deletion mutations in the *COL2A1* gene were identified, accounting for 21 of the 23 families. Different mutations of the *COL2A1* gene were associated with similar phenotypes but with different degrees of expressivity.

## Introduction

Stickler syndrome (MIM #108300) is an inherited connective tissue disorder that affects the eyes, cartilage and articular tissues. The phenotypes of Stickler syndrome include congenital high myopia, retinal detachment, premature joint degeneration, hearing impairment and craniofacial anomalies, such as cleft palate and midline facial hypoplasia.^1^ The disease is caused by mutations of the *COL2A1, COL9A1, COL11A1* or *COL11A2* genes; these genes control the synthesis of type II, type IX and type XI collagens, respectively. Autosomal dominant Stickler syndrome can be caused by mutations in the *COL2A1, COL11A1* or *COL11A2* genes, and an autosomal recessive form is caused by mutations in the *COL9A1* gene.^2,3^ Patients with mutations in the *COL2A1* gene have a characteristic ‘membranous’ or type I vitreous phenotype, and they account for more than 80% of the patients with Stickler syndrome.^4^ Patients with *COL11A1* mutations have a ‘beaded’ or type 2 vitreous phenotype, and patients with *COL11A2* mutations lack any ocular alterations.^1^

The purpose of this study was to determine the phenotype–genotype correlation in Japanese patients with Stickler syndrome caused by mutations in the *COL2A1* gene. To accomplish this, we examined 40 patients from 23 families and identified mutations in the *COL2A1* gene in 21 out of the 23 families.

## Materials and methods

Forty Japanese patients from 23 families with Stickler syndrome were studied. There were 24 men and 16 women whose average age was 22.9±16.2 years, with a range from 1 to 50 years (Table 1). The diagnosis of the disease was based on the criteria presented by Richards *et al.*,^1^ that is, (1) congenital vitreous anomaly and (2) any three of the following abnormalities: high myopia, retinal detachment, paravascular pigmented degeneration, joint degeneration, hearing defect and cleft palate. Of the 23 families, 16 of the cases were familial and 7 were sporadic.

The ocular examinations included measurements of the refractive error, visual acuity and intraocular pressure, and examinations by slit-lamp biomicroscopy and ophthalmoscopy. The presence of systemic diseases was ascertained by answers to a questionnaire, general systemic examinations, and referral letters from orthopedists and otolaryngologists. For the affected family members who could not be examined in person, medical questionnaires were used to determine whether they had retinal detachments.

The study was approved by the Ethics Committee of the University of Occupational and Environmental Health Japan, Fukuoka University, Jikei University and Kinki University Sakai Hospital. Informed consent was obtained from all participants or parents by written signatures.

DNA was extracted from peripheral blood using a DNA extraction kit (QiaAmp, Qiagen, Chatswoth, CA, USA). To identify mutations in the coding exons of the *COL2A1* gene, oligonucleotide primers complementary to flanking introns/untranslated region sequences were designed. PCR was performed and followed by uni- or bi-directional sequencing, depending on the quality of the PCR products. The sequences of the primers and the annealing temperature for PCR for each primer pair are available on request. For deletion mutations, a fragment of the PCR product was cloned, followed by sequencing to differentiate the mutant allele from the wild-type allele using a cloning kit (TOPO TA Cloning Kit, Thermo Fisher Scientific, Waltham, MA, USA) according to the manufacturer’s directions. To identify the sequence variations, a reference sequence of *COL2A1* (NM_001844.4) was used. The variations were numbered based on the corresponding reference complementary DNA sequence, with +1 corresponding to the first nucleotide of the initiation codon (ATG).

For splicing mutations, a splicing site prediction program, NNSPLICE (http://www.fruitfly.org/), was used to determine whether the mutations would abolish the consensus sequences of the splicing sites.

## Results

### COL2A1 mutations

Seventeen different mutations (5 nonsense, 4 splicing and 8 deletion mutations) in the *COL2A1* gene were identified in 21 of the 23 families (91.3%, Table 1). Twelve were novel mutations: NM_001844.4(COL2A1_v001):c.311del, c.762+5G>A, c.1491_1527+11del, c.1522G>T, c.1527+4A>T, c.1963G>T, c.1995+1G>A, c.2077_2078del, c.2539del, c.2818C>T, c.2858del and c.3872_3873del. Five of the mutations have been reported, c.237del, c.1833+1G>A, c.2353C>T, c.2813del and c.3106C>T, according to the Human Gene Mutation Database (https://portal.biobase-international.com/cgi-bin/portal/login.cgi, accessed on Jan 2016). We also found seven sporadic mutations, and two of these mutations were confirmed to be caused by *de novo* mutations. The mutation in family #1, c.237del, was identical to the mutation reported by Yoshida *et al.*^5^

The NNSPLICE program revealed that the c.762+5G>A, c.1995+1G>A and c.1833+1G>A mutations disrupted the consensus sequences of the splicing sites, i.e., the splicing sequences became unpredictable with prediction scores of <0.1 in contrast with scores of >0.96 for the reference sequences. However, it was not clear if c.1527+4A>T aborted the consensus splicing site because both the reference and the mutant sequences of the 5ʹ donor site of intron 23 were not predictable by the NNSPLICE program.

### Clinical symptoms

Ocular and systemic signs and symptoms are shown in Table 1. The most common ocular finding was myopia. All patients had myopia ranging from −2.25 to −18.0 D, with an average of −8.7 diopters at age >1 year. Membranous vitreous degeneration, paravascular pigmented degeneration and cataracts were found in 60 eyes (92.3%, *N*=65), 35 eyes (61.4%, *N*=57) and 26 eyes (35.1%, *N*=74), respectively.

Twenty-two patients (55.0%) had retinal detachment, which was detected at an average age of 15.4 years. Eleven patients had bilateral retinal detachments, and 11 had unilateral detachments. Twenty-eight eyes (84.8%) had a retinal detachment before the age of 20 years (Table 1). Five eyes were phthisical and blind due to retinal detachments in childhood. For the 11 patients who had bilateral retinal detachments, the retinal detachments were diagnosed simultaneously in both eyes for 3 of the 11 individuals. For another 7 of these patients, the interval between diagnoses of retinal detachment in one eye versus the other ranged from 3 to 25 (median 5) years. The answers to the questionnaires indicated that there were 15 additional Stickler patients in 8 families (Families #1, 5, 6, 8, 14, 15, 18 and 23), and 11 of these 15 patients (73.3%) had retinal detachments as well. For six of these 11 patients (54.5%), the retinal detachments were bilateral.

Facial hypoplasia, cleft palate, joint degeneration, and hearing impairment were detected in 20 (50.0%), 16 (40.0%), 9 (22.5%) and 4 (10.0%) patients, respectively.

## Discussion

Twenty-one mutations (91.3%) in the *COL2A1* gene were found in 23 families with Stickler syndromes. All of the mutations were predicted to lead to a premature termination of the gene, resulting in haploinsufficiency of type II collagen.^1^ At present, 196 different mutations in the *COL2A1* gene are known to cause Stickler syndrome according to the HGMD, and we have added 12 new mutations. However, the significance of the c.1527+4A>T mutation could not been determined, and further investigation is needed to determine the effect of this mutation on gene expression or function.

Although mutation hot spots have not been reported, we found recurrent instances of two known mutations, c.1833+1G>A (present in four families) and c.3106C>T (two families). No mutation was found in two families, Families #22 and #23. The proband of Family #22 has the typical signs of type 1 Stickler syndrome and probably has an intronic or severe truncating loss-of-function mutation in the *COL2A1* gene. The proband of Family #23 has an unremarkable type 1 vitreous phenotype and therefore may have a mutation in another gene.

Retinal detachments are a common but serious complication in Stickler syndrome that can lead to blindness in childhood.^4,6^ An effective prophylactic intervention for retinal detachment has not been established, but a recent cryotherapy protocol appears to be effective in preventing the development of retinal detachments in Caucasians.^4^ According to Stickler *et al.*,^7^ who studied 316 patients with Stickler syndrome, 60% of the patients with Stickler syndrome had a retinal detachment, and 4% of them were blind. Comparable frequencies of retinal detachments, 65% (*N*=66), were reported by Parma *et al.*,^8^ with 70% occurring between 4- and 18-years-of-age. Consistent with the aforementioned studies, Donoso *et al.*^9^ reported frequencies of 57% (*N*=165) with an average age of onset of 15 years. Nishimura *et al.*^10^ examined 22 Japanese patients (16 probands) with Stickler syndrome and found 11 mutations (69%) in the *COL2A1* gene. However, only 3 of the 22 patients (27%) had a retinal detachment.^10^ The lower frequency may be because these patients were mainly evaluated by pediatricians and orthopedists rather than by ophthalmologists. In our study, the majority of patients had a mutant *COL2A1* gene, and the incidence of retinal detachments was as high as that reported among Caucasian patients. Moreover, the incidence of retinal detachments from the additional questionnaire was 73.3%.

The presence of facial hypoplasia and a flat nose was also less frequent in Asian patients. Degenerative joints and hearing impairment should be detected more frequently by orthopedists and otolaryngologists.^11^

In summary, mutations in the *COL2A1* gene were found in 21 of 23 Japanese families (91.3%) with Stickler syndrome. Different mutations of the *COL2A1* gene were associated with similar phenotypes, but with different degrees of expressivity. Retinal detachment was the most serious complication, and the incidence was high and comparable to that observed in Caucasian patients. These findings should lead to a better understanding and diagnosis of Stickler syndrome.

## Figures and Tables

**Table 1:** Mutations in the *COL2A1* gene and clinical features in patients with Stickler syndrome

*Patient no.*	*Family no.*	*Kinship*	*Age (years)*	*Sex*	*Familial/sporadic*	*Mutation*				*Ocular features*						*Systemic features*				*Other remarks*
						*Exon/intron*	*Nucleotide change (NM_001844.4)*	*Amino acid change*	*Novel/Reported*	*Refraction (D)*^a^	*Cataract*	*Glaucoma*	*Membranous vitreous*	*Paravascular retinal pigmentation*	*Retinal detachment (onset, year old)*	*Facial hypoplasia*	*Cleft palate*	*Arthropahty*	*Hearing loss*	
1	1	Proband	14	F	Familial	2	c.237del	p.Ile80Serfs*37	Reported	−5/−5.125	+/+	−/−	+/+	+/+	11/−	−	−	−	−	R) V
2		Mother	38	F	Familial	2	c.237del	p.Ile80Serfs*37	Reported	N/N	−/−	−/−	+/+	−/−	−/−	−	−	−	−	
3	2	Proband	10	M	Familial	4	c.311del	p.Gly104Aspfs*13	Novel	−14/N	N/+	N/−	N/+	N/−	3/9	−	+	−	−	R) phthisis
4		Mother	35	F	Familial	4	c.311del	p.Gly104Aspfs*13	Novel	−5.5/−4.5	+/IOL	−/−	+/+	N/N	35/−	−	−	−	−	
5		Sister	4	F	Familial	4	c.311del	p.Gly104Aspfs*13	Novel	−9/−8	−/−	−/−	+/+	N/N	−/−	−	+	−	−	B) megalocornea
6		Grandfather	63	M	Familial	4	c.311del	p.Gly104Aspfs*13	Novel	N/−11	N/+	N/−	N/N	N/−	8/30	−	−	−	−	R) phthisis
7		Uncle	50	M	Familial	4	c.311del	p.Gly104Aspfs*13	Novel	−10/−5	+/+	−/−	N/N	N/N	−/−	−	−	−	−	B) IOL, Chorioretinal atrophy
8	3	Proband	17	M	Sporadic	11	c.762+5G>A	USE	Novel	−14/−10	+/+	−/−	+/+	N/N	14/17	+	−	+	+	R) ENC,V,IOL, L) ENC
9	4	Proband	15	M	Sporadic	23	c.1491_1527+11del	p.Gly498Trpfs*28	Novel	N/N	−/−	−/−	−/−	+/+	10/15	+	−	−	−	B) V
10	5	Proband	36	M	Familial	23	c.1522G>T	p.Glu508*	Novel	−18/N	N/−	N/+	N/+	N/+	5/30	−	−	−	−	R) phthisis, L) IOL
11	6	Proband	20	M	Familial	23	c.1527+4A>T	USE	Novel	−7/−6	−/−	−/−	+/+	−/−	−/−	−	−	−	−	
12	7	Proband	18	M	Sporadic	27	c.1833+1G>A	USE	Reported	−3.5/−5.25	−/−	−/−	+/+	+/+	16/−	+	+	+	+	R) V,IOL
13	8	Proband	14	M	Familial	27	c.1833+1G>A	USE	Reported	−6.5/−6.25	−/−	−/−	+/+	−/−	12/−	+	−	−	+	L) V,IOL
14	9	Proband	12	F	Familial	27	c.1833+1G>A	USE	Reported	−7.75/−7.75	−/−	−/−	+/+	+/+	−/−	+	+	+	−	−
15		Sister	14	F	Familial	27	c.1833+1G>A	USE	Reported	−2.25/−2.75	−/−	−/−	+/−	+/+	−/−	+	−	−	−	−
16		Mother	35	F	Familial	27	c.1833+1G>A	USE	Reported	−6.625/−3.75	−/−	−/−	+/+	−/−	−/−	+	−	+	−	−
17	10	Proband	10	M	Familial	27	c.1833+1G>A	USE	Reported	−11/−11	+/+	−/−	+/+	N/N	9/9	−	−	−	−	
18		Mother	38	F	Familial	27	c.1833+1G>A	USE	Reported	−8/−9	+/+	−/−	+/+	N/N	40/40	−	−	−	−	
19	11	Proband	37	F	Familial	30	c.1963G>T	p.Glu655*	Novel	−11/−12.25	−/−	−/−	+/+	+/+	−/−	+	−	−	−	
20	12	Proband	27	M	Sporadic	30	c.1995+1G>A	USE	Novel	−8/N	−/−	−/−	+/+	+/+	7/−	+	+	+	−	R) phthisis
21	13	Proband	10	M	Familial	32	c.2077_2078del	p.Gly693Profs*7	Novel	−14/−12.75	−/−	−/−	+/+	−/−	7/−	−	+	−	−	
22		Mother	40	F	Familial	32	c.2077_2078del	p.Gly693Profs*7	Novel	−10.75/−13	−/−	−/−	+/+	−/−	11/15	+	+	−	−	
23	14	Proband	10	M	Familial	35	c.2353C>T	p.Arg785*	Reported	−6/−6.75	−/−	−/−	+/+	+/+	10/−	+	+	N	−	
24		Brother	1	M	Familial	35	c.2353C>T	p.Arg785*	Reported	N/N	N/N	N/N	N/N	N/N	−/−	+	+	−	−	Acute respiratory distress syndrome
25		Sister	5	F	Familial	35	c.2353C>T	p.Arg785*	Reported	−10/−10	−/−	−/−	+/+	N/N	−/−	+	+	−	−	
26	15	Proband	39	F	Familial	39	c.2539del	p.Ala847Profs*34	Novel	−14/−10	+/+	−/−	+/+	+/+	−/−	+	+	+	−	
27	16	Proband	13	M	Sporadic	42	c.2813del	p.Pro938Leufs*90	Reported	−5.25/−4.65	−/−	−/−	+/+	+/+	−/−	+	−	−	+	
28	17	Proband	10	M	Familial	42	c.2818C>T	p.Arg940*	Novel	−14.125/−15.5	−/−	−/−	+/+	+/+	10/−	−	+	−	−	
29		Father	45	M	Familial	42	c.2818C>T	p.Arg940*	Novel	−5.25/−4.625	−/−	−/−	N/N	+/+	−/−	−	−	−	−	
30	18	Proband	15	M	Familial	42	c.2858del	p.Pro953Leufs*75	Novel	−11.25/−10	−/IOL	−/−	N/N	−/−	14/−	−	−	+	−	L) V
31		Mother	47	F	Familial	42	c.2858del	p.Pro953Leufs*75	Novel	−15/N	IOL/IOL	−/N	N/N	−/−	26/N	−	−	−	−	B) V, L) phthisis
32	19	Proband	10	M	Familial	44	c.3106C>T	p.Arg1036*	Reported	−7/−5	−/−	−/−	N/+	+/+	8/−	−	−	−	−	
33		Mother	35	F	Familial	44	c.3106C>T	p.Arg1036*	Reported	−8/−9	+/+	−/−	+/+	+/+	−/−	−	−	−	−	
34	20	Proband	3	F	Familial	44	c.3106C>T	p.Arg1036*	Reported	−5/−5	−/−	−/−	+/+	−/−	−/−	+	+	−	−	
35		Sister	3	F	Familial	44	c.3106C>T	p.Arg1036*	Reported	−6.5/−9	−/−	−/−	+/+	−/−	−/−	+	+	−	−	
36		Father	49	M	Familial	44	c.3106C>T	p.Arg1036*	Reported	−9/N	+/+	−/−	+/+	+/+	15/−	−	−	−	−	
37	21	Proband	18	F	Sporadic	51	c.3872_3873del	p.Pro1291Argfs*10	Novel	−18/−16	+/+	−/−	+/+	N/N	14/18	−	−	−	−	
38	22	Proband	12	F	Sporadic	—	ND	ND		−6/−4.5	+/+	−/−	−/−	−/−	12/12	+	+	+	−	B) ENC
39	23	Proband	5	M	Familial	—	ND	ND		−11.5/−10.3	−/−	−/−	+/+	+/+	−/−	+	+	+	−	
40		Father	37	M	Familial	—	ND	ND		−9.5/−3.75	−/−	−/−	+/+	+/+	−/−	+	−	−	−	

Abbreviations: B, both eyes; D, diopters; ENC, encircling surgery; F, female; IOL, intraocular lens; L, left eye; M, male; N, not described (determined); ND, mutation not detected; R, right eye; USE, undetermined splicing effect; V, vitreous surgery.

a Negative values indicate myopia, and only preoperative refractions are shown if cataract surgery was performed.
